# The Growth of *Eimeria tenella*: Characterization and Application of Quantitative Methods to Assess Sporozoite Invasion and Endogenous Development in Cell Culture

**DOI:** 10.3389/fcimb.2020.579833

**Published:** 2020-10-08

**Authors:** Virginia Marugan-Hernandez, Georgia Jeremiah, Kelsilandia Aguiar-Martins, Alana Burrell, Sue Vaughan, Dong Xia, Nadine Randle, Fiona Tomley

**Affiliations:** ^1^The Royal Veterinary College, University of London, London, United Kingdom; ^2^Electron Microscopy Science Technology Platform, The Francis Crick Institute, London, United Kingdom; ^3^Department of Biological and Medical Sciences, Oxford Brookes University, Oxford, United Kingdom; ^4^Department of Infection Biology, Institute of Infection & Global Health, University of Liverpool, Liverpool, United Kingdom

**Keywords:** *Eimeria tenella*, cell culture, endogenous development, electron microscopy, quantitative PCR, anticoccidial inhibition

## Abstract

*In vitro* development of the complete life cycle of *Eimeria* species has been achieved in primary cultures of avian epithelial cells with low efficiency. The use of immortalized cell lines simplifies procedures but only allows partial development through one round of parasite invasion and intracellular replication. We have assessed the suitability of Madin-Darby Bovine Kidney (MDBK) cells to support qualitative and quantitative studies on sporozoite invasion and intracellular development of *Eimeria tenella*. Analysis of parasite ultrastructure by transmission electron microscopy and serial block face—scanning electron microscopy proved the suitability of the system to generate good quality schizonts and first-generation merozoites. Parasite protein expression profiles elucidated by mass spectrometry corroborated previous findings occurring during the development of the parasite such as the presence of alternative types of surface antigen at different stages and increased abundance of proteins from secretory organelles during invasion and endogenous development. Quantitative PCR (qPCR) allowed the tracking of development by detecting DNA division, whereas reverse transcription qPCR of sporozoite- and merozoite-specific genes could detect early changes before cell division and after merozoite formation, respectively. These results correlated with the analysis of development using ImageJ semi-automated image analysis of fluorescent parasites, demonstrating the suitability and reproducibility of the MDBK culture system. This systems also allowed the evaluation of the effects on invasion and development when sporozoites were pre-incubated with anticoccidial drugs, showing similar effects to those reported before. We have described through this study a series of methods and assays for the further application of this *in vitro* culture model to more complex studies of *Eimeria* including basic research on parasite cell biology and host-parasite interactions and for screening anticoccidial drugs.

## Introduction

*Eimeria tenella* is a host- and tissue-specific parasite, replicating *in vivo* only in the epithelial cells that line the caeca of the domestic chicken. This parasite together with six other species (*E. acervulina, E. brunetti, E. maxima, E. mitis, E. necatrix*, and *E. praecox*) is responsible for chicken coccidiosis, an enteric disease characterized by malabsorption, diarrhea, and/or hemorrhage with a significant impact on chicken meat and egg production worldwide. Unlike other *Eimeria* species that infect chickens, purified sporozoites of *E. tenella* invade and undergo some endogenous development in avian and mammalian cells *in vitro*. There was early optimism that avian cells might support the whole developmental life cycle when small numbers of male and female gametocytes were observed several days following sporozoite infection (Strout and Ouellette, 1969). However, despite many efforts and technical innovations the best results achieved to date have come from using primary embryonic chick kidney cells where small numbers of unsporulated oocysts can be produced (~10^2^ oocysts from an inoculation of 10^4^ to 10^5^ sporozoites), which sporulate very poorly compared to oocysts produced in chickens (Doran, 1971; Doran and Augustine, 1973; Hofmann and Raether, 1990).

The use of immortalized mammalian cell lines, which are simpler to maintain than primary avian cells, has proven useful to study the early part of the *E. tenella* developmental cycle. Non-avian cells support a single round of asexual growth (schizont development and the release of first-generation merozoites) and for this they are at least as efficient as primary chick cell cultures (Patton, 1965). Several studies agree that many mammalian cell lines are susceptible to sporozoite invasion however they differ widely in their capacity to support schizogony and merozoite formation (Doran and Vetterling, 1967; Tierney and Mulcahy, 2003). The most reliable cells used to date for the study of *E. tenella* sporozoite invasion and intracellular growth are Madin-Darby Bovine Kidney (MDBK) epithelial cells (Brown et al., 2000; Bumstead and Tomley, 2000; Tierney and Mulcahy, 2003). These have been used to examine phenotypes of transgenic *E. tenella* parasites generated by transfection (Clark et al., 2008; Marugan-Hernandez et al., 2017b) and to test the impacts of anticoccidial drugs (Zhu et al., 1994; Jenkins et al., 2014; Thabet et al., 2015, 2017), antibodies (Whitmire et al., 1988), cytokines (Kogut and Lange, 1989), natural compounds (Allen, 2007; Alnassan et al., 2015), and competition with other organisms (Tierney et al., 2004).

In this study, we have used this MDBK cell culture system to develop reliable quantitative *in vitro* assays that measure parasite replication and gene expression during the first round of intracellular schizogony of *E. tenella*. The assays incorporate the use of quantitative PCR (qPCR) as well as reverse transcription qPCR (RT-qPCR) that targets expression of stage-specific (sporozoite and merozoite) genes, combined with semi-automated image analysis. We demonstrate the utility of these combined assays for development of high throughput *in vitro* systems to evaluate anti-parasitic effects of novel treatments and identify by mass spectrometry potential targets expressed at different time points during first generation schizogony.

## Materials and Methods

### Parasites and Birds

The *E. tenella* Wisconsin (Wis) strain (McDougald and Jeffers, 1976) and a transgenic population, *E. tenella* YFPmYFP, derived from this strain (Clark et al., 2008) were propagated in 3-week-old White Leghorn chickens reared under specific pathogen free conditions as previously described (Shirley, 1995). Oocyst purification, excystation and sporozoite purification were performed as described previously (Pastor-Fernandez et al., 2019). Freshly purified sporozoites were used to infect cell monolayers.

### Cell Culture

The NBL-1 line of MDBK cells (NBL-1; ECACC-Sigma-Aldrich, Salisbury, UK) was used throughout. Cells were passaged twice weekly by trypsinization of confluent monolayers and maintained in T75 (10 × 10^6^ cells/well) flasks at 37°C, 5% CO_2_ in Advanced DMEM (Gibco, Leicestershire, UK) supplemented with 2% fetal bovine serum (FBS; Sigma, Suffolk, UK) and 100 U/ml penicillin/streptomycin (Fisher, Leicestershire, UK). For sporozoite infections, freshly trypsinized MDBK cells were seeded into 24-well plates (0.3 × 10^6^ cells/well).

### *In vitro* Endogenous Development

Freshly seeded MDBK monolayers were infected with sporozoites (1 × 10^6^ sporozoites/well, 3 h after seeding) and incubated at 41°C, 5% CO_2_. At 4, 20, 24, 28, 44, 48, 52, and 68 h post infection (hpi) infected monolayers (3 wells/time point, technical replicates) were recovered by pipetting into 0.35 ml of RTL buffer (Qiagen, West Sussex, UK) and stored at −20°C. These experiments were performed in triplicate for both *E. tenella* Wis (three biological replicates) and *E. tenella* YFPmYFP (three biological replicates). Alternately, monolayers (3 wells/time point, technical replicates) were washed gently in phosphate-buffered saline (PBS; 3 × 1 ml) and fixed in 3% paraformaldehyde in PBS solution followed by a further wash in PBS and storage at 4°C. This experiment was performed in triplicate for *E. tenella* YFPmYFP (three biological replicates).

### Anticoccidial Drugs Assay

Sporozoites (1 × 10^6^ per replicate) of *E. tenella* Wis strain were pre-incubated for 1 h at 41°C, 5% CO_2_ with a selection of anticoccidial compounds [amprolium (AMP), robenidine (ROB), and salinomycin (SAL)] or with cytochalasin D (CYT); all compounds were suspended to a final concentration of 5 μg/ml in PBS just before use, made from stock concentrations of 10 mg/ml in dimethyl sulfoxide (DMSO). DMSO alone was also included as a control (0.05% final volume). After incubation, sporozoites were washed with PBS, resuspended in DMEM and added to MDBK monolayers (3 wells/time point/condition, technical replicates). After 2, 24, 44, and 52 hpi cells were recovered using 0.35 ml of RTL buffer (Qiagen) for further DNA extraction. This experiment was performed in duplicate for *E. tenella* Wis (two biological replicates).

### Isolation of Nucleic Acids and Synthesis of Complementary DNA (cDNA)

Genomic DNA (gDNA) and RNA were isolated from the samples stored in RTL buffer (Qiagen) using the AllPrep DNA/RNA Mini Kit (Qiagen) following manufacturer's instructions. Complementary DNA (cDNA) was synthesized from total RNA following the procedure described by Marugan-Hernandez et al. (2016).

### Real Time Quantitative PCR (qPCR) and Reverse Transcription qPCR (RT-qPCR)

Real time quantitative PCR (qPCR) was performed in a CFX96 Touch® Real-Time PCR Detection System (Bio-Rad, Hertfordshire, UK) following the procedures described previously (Marugan-Hernandez et al., 2016). For parasite quantification, the number of haploid genomes (equivalent to single sporozoites or merozoites) per well (3 wells/sample, technical replicates) was determined for each time point using gDNA specific primers for *Eimeria* spp. 5S rDNA (Fw_5S: TCATCACCCAAAGGGATT, Rv_5S: TTCATACTGCGTCTAATGCAC) (Clark et al., 2008) and a standard curve of sporozoite gDNA extracted by the same methods.

For the evaluation of the endogenous development, transgene transcription was quantified from cDNA (RT-qPCR) using specific primers for the sporozoite (SP25: Fw_SP25: AGGCTCTTTACTATGTCCA, Rv_SP25: CAAAAAACACATACAGACGC) and merozoite-specific (MZ80: Fw_MZ80: TTTCGCCGCATGATCATAT, Rv_MZ80: CGATGTCTCCTCTCCAATT) genes from the *esf2* family described by Reid et al. (2014); together with actin (Fw_Actin: TTGTTGTGGTCTTCCGTCA, Rv_Actin: GAATCCGGGGAACATAGTAG). Transcript levels were compared with serial dilutions of DNA standard templates for each transgene (pGEM®-T Easy plasmid (Promega, Hampshire, UK) containing the SP25, MZ80 or actin coding sequences). Transcript numbers along a time-course of intracellular schizont development were normalized to the number of parasite genomes. Data were analyzed with the Bio-Rad CFX Manager software (Bio-Rad).

### Light and Fluorescent Microscopy

Digital images from fixed cell monolayers infected with fluorescent *E. tenella* YFPmYFP along the endogenous development time course were captured using a Leica DMI300 B microscope equipped with a high-speed camera DCF365FX (Leica Microsystems, Milton Keynes, UK). Transgenic *E. tenella* YFPmYFP expressing the yellow fluorescent protein (YFP) were detected with the fluorescein isothiocyanate filter (FITC). Exposure, gain, gamma and resolution were adjusted remotely through the Leica application suite software v4.0.0.

### ImageJ Semi-Automated Image Analysis

Semi-automated image analysis of representative micrographs taken at each stage during the infection time-course (4, 20, 24, 28, 44, 48, 52, and 68 hpi) was accomplished using a custom script designed to utilize existing ImageJ components, implement new functionality and greatly increase data output.

Digital images of a given resolution comprise a fixed number of pixels determined by their dimensions. Each pixel is assigned a value ranging from 0 to 255 in an 8-bit image, dependent upon the intensity of light detected by the microscope camera at the time of capture. The original pixel values are altered when color channels are applied, leading to an unwanted loss of data integrity. Therefore, each captured image was stored as an 8-bit depth TIF file for continuity purposes and lookup tables (LUTs) were applied to give the illusion of color without altering core pixelation data. Using the ImageJ scripting language, a program was created to load an image, apply LUTs, identify and threshold structures of interest (sporozoite or schizont), mask, map and measure each structure before summarizing individual perimeter and area data.

A LUT is a customizable color map, in which a user can define an array of display colors for given grayscale values between 0 and 255. LUTs allow the user to distinguish between the two different structures with similar light intensities and the “Green Fire Blue” LUT was applied to loaded images, staining sporozoites bright green and schizonts deep blue. Threshold values were hard-coded to maintain consistency between images. Threshold masks were applied separately to duplicate images for sporozoites and schizonts before being converted to binary images with two possible pixel values—black and white. Particle analysis was then conducted by identifying edges at the juxtaposition of white and black pixels to produce structure outlines, record perimeter data and calculate the particle area using ImageJ's inbuilt mathematical library. Perimeter and area data collected from processed images was recorded for analysis.

### Samples for Election Microscopy

One-hundred microliters of 25% electron microscopy grade glutaraldehyde were added to MDBK monolayers after 48 hpi in 1 ml of cell-culture medium. Fifteen minutes after the addition of glutaraldehyde, cell-culture medium was removed and replaced by 1 ml of primary fix [20% freshly-prepared formaldehyde solution (10%), 10% electron microscopy grade glutaraldehyde (25%), and 50% sodium cacodylate buffer (0.2 M) in double distilled (dd)H_2_O]. Monolayers were washed five times in 0.1 M cacodylate buffer followed by a 60 min incubation in 500 μl of 4% osmium tetroxide and 500 μl of 0.2 M sodium cacodylate buffer. Fixed cells were washed five times in ddH_2_O followed by an overnight incubation in 2% freshly-filtered aqueous uranyl acetate (at 4°c and in the dark). Cells were washed three times in ddH_2_0. Cells were then dehydrated by a series of 20 min incubations in increasing concentration of ethanol in ddH_2_0. Dehydration steps consisted of 30% ethanol, 50% ethanol, 70% ethanol, 90% ethanol, and 100% ethanol. One milliliter of propylene oxide (TAAB) was added to each well and left for 30 s. Agitating the solution using a Pasteur pipette resulted in detachment of the monolayer which could then be transferred to a 1.5 ml microcentrifuge tube. Detached monolayers were then incubated in three changes of 100% anhydrous acetone, each for 10 min. Cells were incubated in 25% TAAB 812 resin in acetone for 2 h, 50% resin overnight; 75% resin for 6 h; 100% resin overnight and two changes of 100% resin, each for 2 h. Infected-monolayer pellets were centrifuged for 20 min in fresh 100% resin and polymerized by incubation at 60°C for 24 h.

### Transmission Electron Microscopy (TEM)

All two-dimensional electron microscopy imaging was performed using a Hitachi™ H-7560 transmission electron microscope with bottom mount camera (2k CCD, Advanced Microscopy Techniques) and manual tilt and rotation capabilities. Digital images were captured using either 1 or 4 integrations depending on sample stability.

### Serial Block Face—Scanning Electron Microscopy (SBF-SEM)

SBF-SEM data was collected using a Zeiss™ Merlin scanning electron microscope with Gatan™ 3View™ automated sectioning and image capture system. The block-face was imaged using a scanning electron beam with 3–5 kV accelerating voltage. Pixel size was between 3 and 7 nm with a section thickness from 60 to 100 nm. Dwell time was ~2 s per pixel with a scan area of around 4,000 × 4,000 pixels. SBF-SEM data was processed using IMOD™ software, run through Cygwin™ command line interface.

### Mass Spectrometry (MS)

MDBK cells infected with *E. tenella* sporozoites at a 4:1 in ratio in T25 flasks (two per time point to generate enough material) were harvested at 4, 24, 36, and 48 hpi. Three biological replicates were prepared per time point. Sample preparation, trypsin digestion and analysis using an LCMS/MS system comprising an Ultimate 3000 nano system coupled to a Q-Exactive mass spectrometer (Thermo Fisher Scientific, Waltham, MA, USA) was performed as described by Horcajo et al. (2018). Spectral data were transformed into.mgf files with Progenesis QI and exported for peptide identification using the Mascot (version 2.3, Matrix Science, London, UK) search engine and the database ToxoDB-26 (version 26, ToxoDB). Protein abundance (iBAQ) was calculated as the sum of all the peak intensities (from the Progenesis output) divided by the number of theoretically observable tryptic peptides for a given protein. Heat maps from MS data were generated Morpheus software (https://software.broadinstitute.org/morpheus/) using hierarchical clustering (one-minus Pearson correlation) to cluster proteins showing similar patterns along the intracellular development.

### Data Analysis

Dataset distributions were ascertained using Shapiro-Wilk and D'Agostino-Pearson omnibus normality tests. The skewed 5S, SP25, MZ80, and actin qPCR/RT-qPCR datasets were analyzed using a Friedman's test for each set of triplicate repeats per time point, whilst normally distributed data obtained from the custom ImageJ script were analyzed using a repeated measures one-way ANOVA with a Geisser-Greenhouse correction. Comparisons across contrasting datasets were first normalized (0% = smallest mean, 100% = largest mean per dataset) before using parametric Pearson Coefficient or non-parametric Spearman's Rank tests based on the presence or absence of a Gaussian distribution. Statistically significant differences were established using a *p* < 0.05 and all analyses, including calculation of arithmetic parameters and normalization of comparative datasets were performed using Graphpad Prism software v7.0b (California, USA).

The normality of data for the anticoccidial drug assay was evaluated using Kolmogorov-Smirnov test. Comparison of groups evaluating one factor was performed using One-way Anova for parametric data and Kruskal-Wallis test for non-parametric. Both analyses were followed by Dunnett's multiple comparisons as a *post-hoc* test using Graph Pad Prism 8.0 (California, USA) or SPSS Statistics 22 (New York, USA). Two-way Anova was used to compare two factors among groups. The relative level of inhibition of *E. tenella* between groups treated with anticoccidial drugs was additionally assessed by a method adapted from Thabet et al. (2017). For this, the proportion of invasion or reproduction of parasites was calculated by normalizing samples to DMSO controls to characterize the inhibition level:

$$\begin{array}{l} \text{Level of Inhibition }\left(\text{\%}\right)=100*\left(1-\frac{\text{Average number of E}.\text{ tenella genomes in treated sample}}{\text{Average number of E}.\text{ tenella genomes in sample treated with DMSO}}\right) \end{array}$$

### Ethical Statement

This study was carried out in strict accordance with the Animals (Scientific Procedures) Act 1986, an Act of Parliament of the United Kingdom. All animal studies and protocols were approved by the Royal Veterinary College Ethical Review Committees and the United Kingdom Government Home Office under specific project licenses. The laboratory work involving GMO was conducted under authorization GM9708.1, administered by the UK Health and Safety Executive. The animal facilities for GMO are classified derogated containment level 3.

## Results

### *In vitro* MDBK Cell Culture Supported the Endogenous Development of *E. tenella* Leading to Fully Formed First-Generation Merozoites

**Figure 1:**
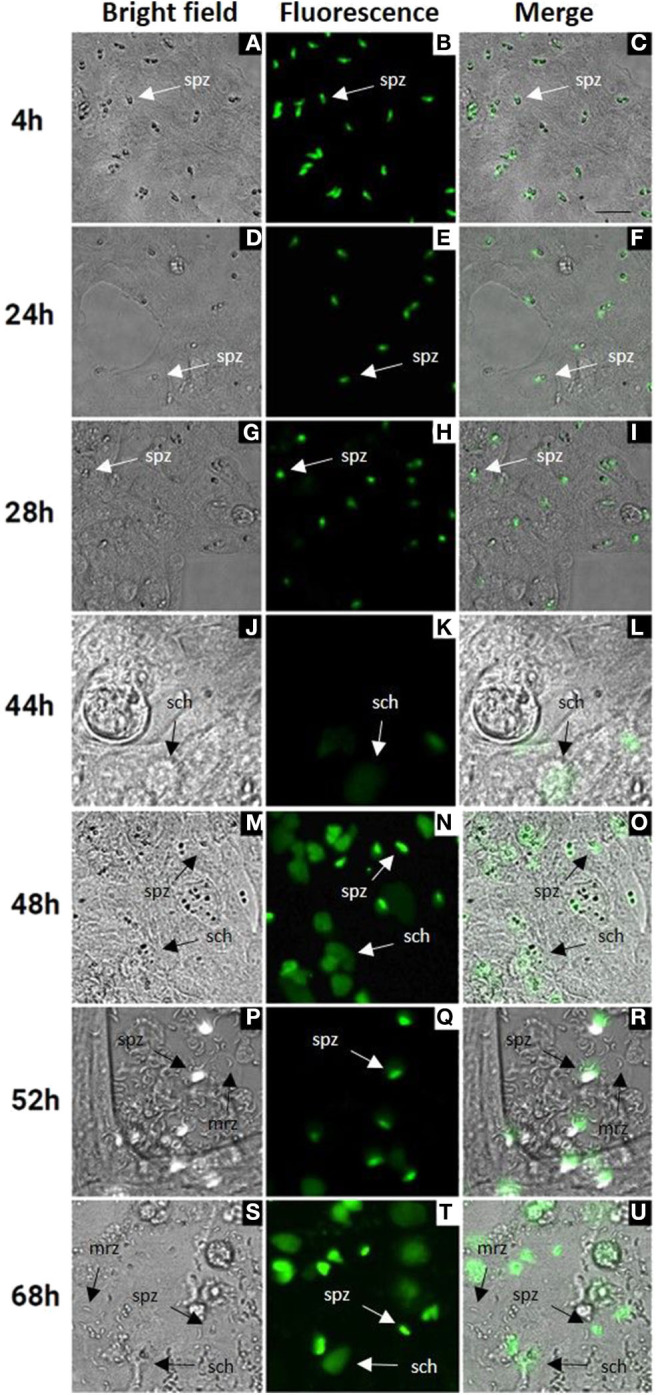
MDBK cell monolayers after infection with *E. tenella* YFPmYFP sporozoites. Time points after infection are indicated in the left side (4–68 h). **(A–C)** Sporozoites (spz) after invasion of MDBK cells. **(D–I)** Rounded sporozoites (spz) preparing to undergo schizogony. **(J–L)** Appearance of early schizonts (sch). **(M–O)** Development of late schizonts (sch) with presence of undeveloped sporozoites (spz). **(P–U)** Release of first-generation merozoites (mrz) with presence of late schizonts (sch) and undeveloped sporozoites (spz), cell monolayer destruction is observed. Scale ~25 μm.

Cell culture conditions were optimized for an optimal rate of invasion and development of *E. tenella* sporozoites without damaging the MDBK monolayers. In monolayers with over 70% of infected cells (Figure 1A) parasites were left for spontaneous development into schizonts. Since first generation schizont development of *E. tenella* in cell culture (from sporozoite infection to the release of first-generation merozoites) is not easily visualized by bright field, we used transgenic parasites expressing a tandemly inserted double copy of YFP under control of the 5'UTR of the *E. tenella* microneme gene *EtMIC1* (*E. tenella* YFPmYFP; Clark et al., 2008) to track parasite development *in vitro*. Micrographs of bright field supported by fluorescence microscopy at different time points (Figure 1) showed the progressive appearance and development of mature schizonts from 44 h post infection (hpi) (Figures 1M–O) until the release of first-generation merozoites from 52 hpi (Figures 1P–U). Since YFPmYFP is regulated by the EtMIC1 promoter mature merozoites showed a reduced fluorescence (Marugan-Hernandez et al., 2017b).

**Figure 2:**
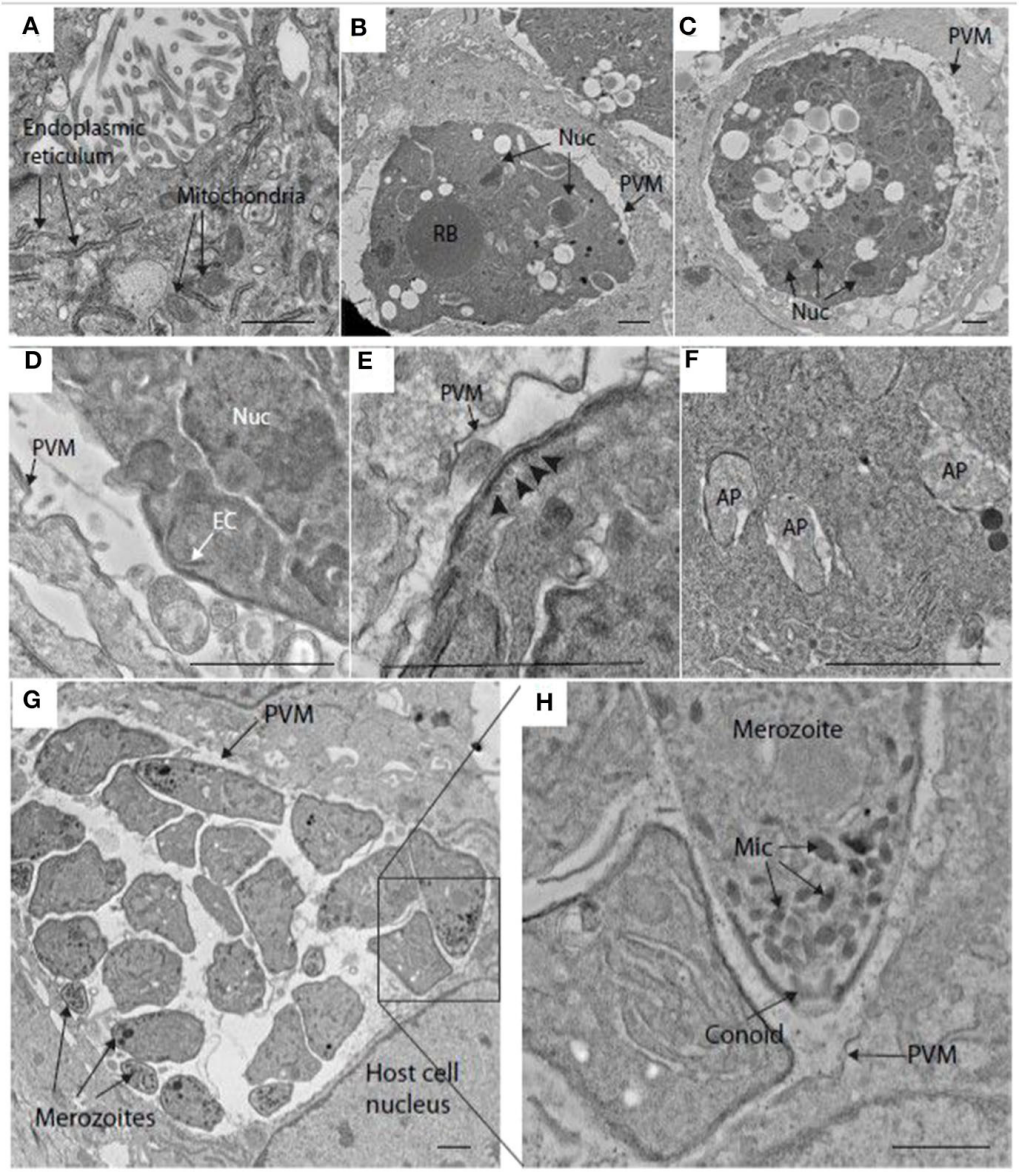
Transmission electron microscopy (TEM) image of schizonts in MDBK cells. **(A)** MDBK cell at 48 hpi showing mitochondria and endoplasmic reticulum. **(B,C)** Parasite at 48 hpi containing multiple nuclei (Nuc) within a specialized compartment bound by the parasitophorous vacuole membrane (PVM). **(D)** Early merozoite formation at the surface of a schizont at 48 hpi. A structure resembling an early conoid (EC) is near one of the schizont nuclei (Nuc). **(E)** Surface of a schizont close to the PVM, microtubules can be seen running below the multi-laminar schizont surface (arrowheads, 48 hpi). **(F)** Multiple apicoplasts (AP) within the cytoplasm of a schizont at 48 hpi. **(G)** Mature schizont encased within a PVM containing several well-developed merozoites at 68 hpi (some indicated by arrows). **(H)** This image is a magnification form the squared area in image **(G)**. showing some merozoite organelles such as the micronemes (Mic) and conoid. Scale ~1 μm.

Transmission electron microscopy (TEM) confirmed the presence of schizonts within MDBK cells and provided fine detail for some of the structures. For example, at 48 h post-infection, host cell endoplasmic reticulum and mitochondria were easily visualized in MDBK cells (Figure 2A). At this time, maturing schizonts containing multiple nuclei were observed within parasitophorous vacuoles, delineated by a parasitophorous vacuole membrane (Figures 2B,C). Immature merozoite structures were visible including newly formed conoid structures that could be seen budding from the periphery of some schizonts (Figure 2D) as well as intra-schizont structures including microtubules, situated just under the schizont multi-layered membrane and several multi-laminar apicoplasts (Figures 2E,F). Mature schizonts were also visualized by TEM at 68 hpi revealing the presence of multiple fully formed first generation merozoites contained within the parasitophorous vacuole membrane (Figure 2G). Inside the merozoites, apical complex structures including micronemes and fully formed conoids (Figure 2H) were seen. These observations confirmed the suitability of this *in vitro* cell system to support the development of good quality schizonts and first-generation merozoites.

**Figure 3:**
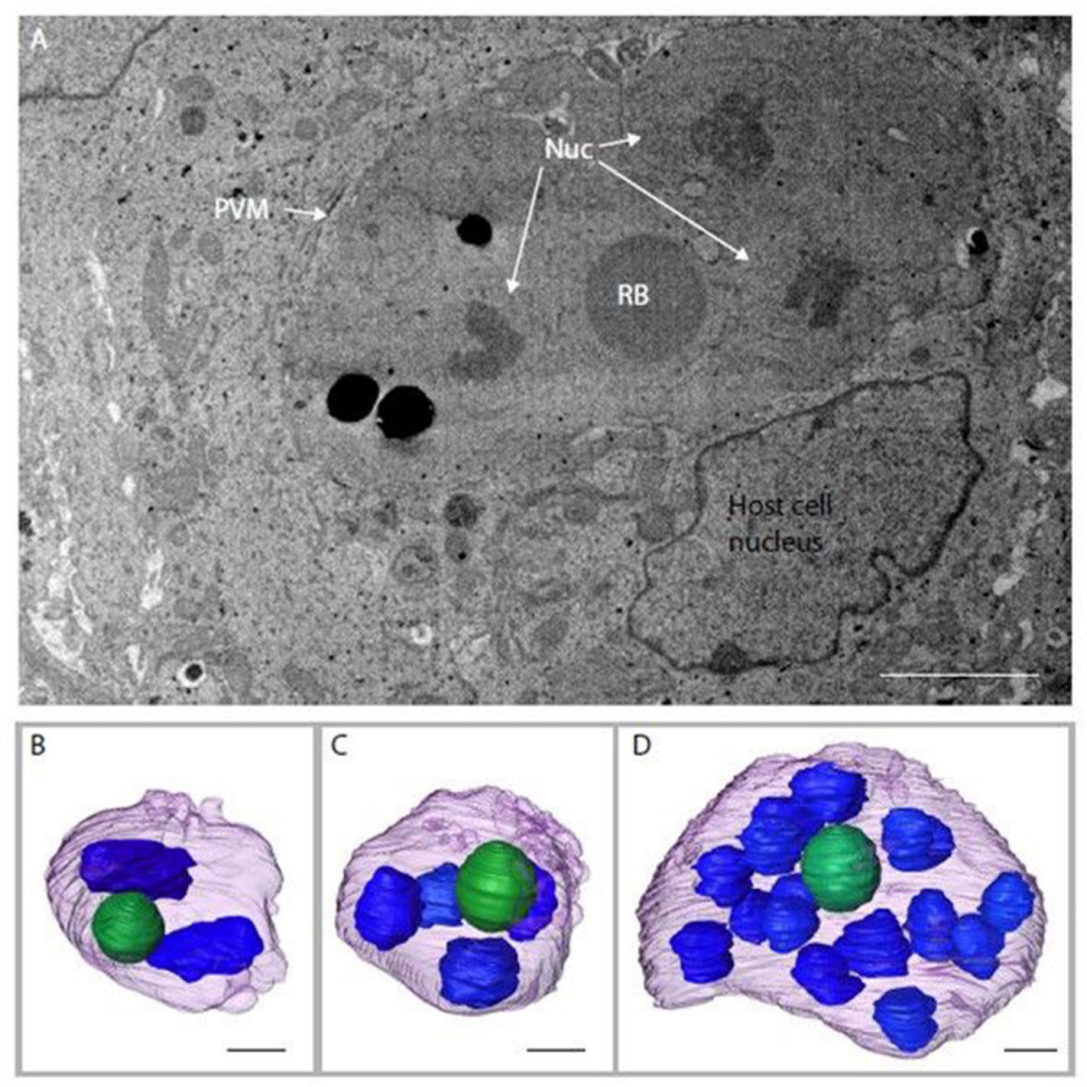
Schizonts imaged by SBF-SEM. **(A)** Single slice from SBF-SEM data showing a multi-nuclear schizont developing within a host cell at 48 hpi (Nuc, nuclei; RB, refractile body; PVM, parasitophorous vacuole membrane). **(B-D)** Surface-rendered models of three schizonts found by SBF-SEM imaging at 48 hpi: schizont with a volume of 311.4 μm^3^ containing two nuclei (blue) and a single refractile body (green) **(B)**; schizont with a volume of 308.7 μm^3^ containing four nuclei and a single refractile body **(C)**; schizont with a volume of 359.8 μm^3^ containing 16 nuclei and a single refractile body **(D)**. Schizont outer surface is shown in purple. Scale ~2 μm.

Serial block face-scanning electron microscopy (SBF-SEM) was carried out to obtain sequential serial sections of whole individual MDBK cells containing intracellular parasites. A total of 836 sequential serial sections were captured and vacuoles containing schizonts were identified. The parasitophorous vacuole membrane, schizont nuclei and single refractile body were segmented for each schizont (Figure 3A). Of the three schizonts imaged by this technology, one of the schizonts contained two nuclei, one contained four nuclei and one contained 16 nuclei (Figures 3B–D). As these nuclei numbers are all within a doubling sequence [2, 4, (8), 16] this data suggests co-ordinated nuclear division, however, further analysis is required to assess this hypothesis.

### Differential Expression of Proteins Related to Invasion and Endogenous Development Processes Can Be Detected in the MDBK *in vitro* System

**Figure 4:**
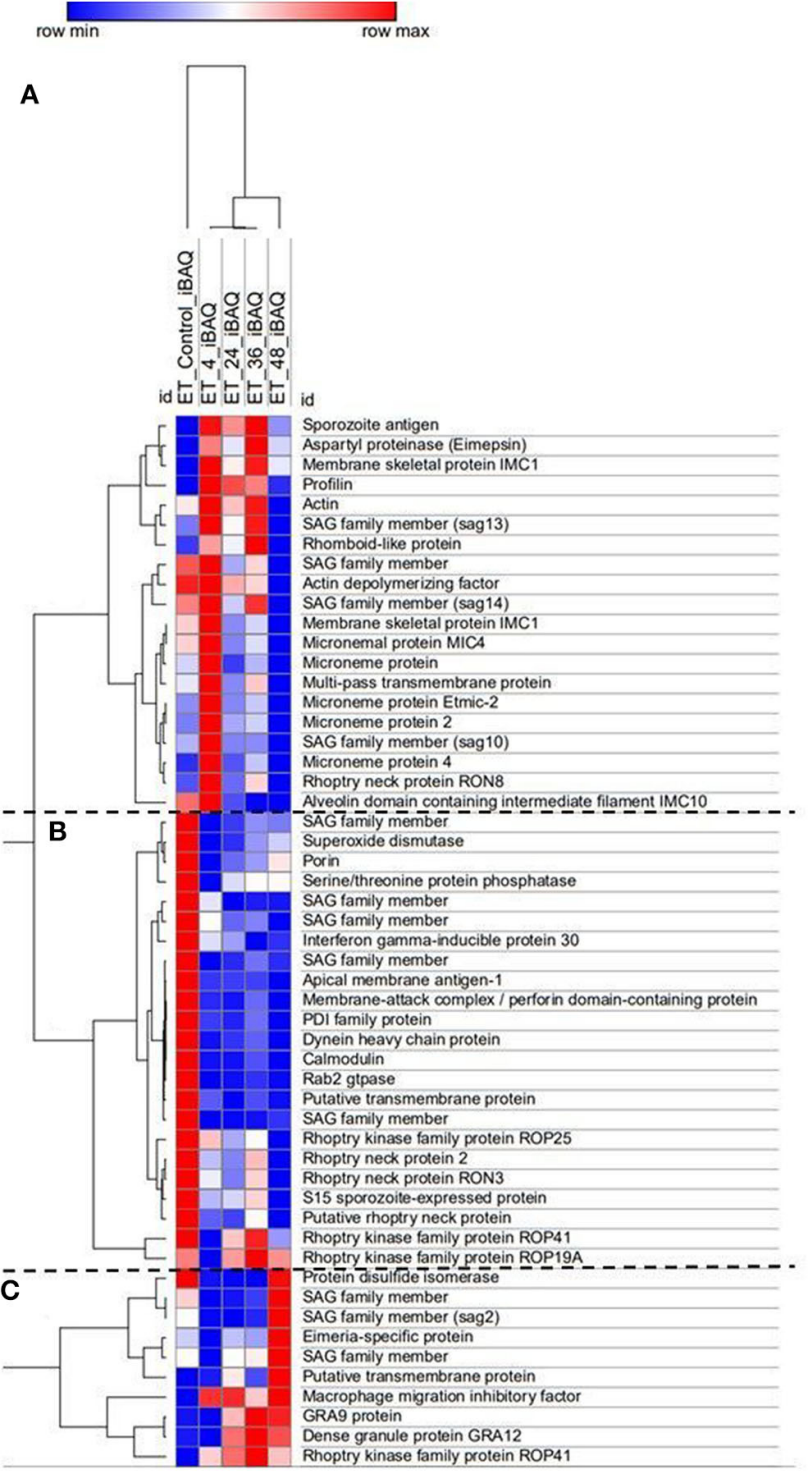
Heat map of *E. tenella* proteins differentially expressed during the *in vitro* development with samples taken at 4, 24, 36, and 48 hpi and freshly-purified sporozoites (control). Cluster **(A)** Proteins highly expressed early after invasion (4 hpi). Cluster **(B)** Proteins expressed in sporozoites whose expression decreased right after invasion and development. Cluster **(C)** Proteins highly expressed during late schizont development (36–48 hpi) and fully-formed first-generation merozoites (48 hpi). iBAQ = Protein abundance. Red/Blue = high/low expression of an individual protein in one specific time point in relation with the rest of time course point and control. Shades of Red/Blue = intermediate values of expression in relation to the extreme values (Red/Blue).

Over 200 proteins were identified by MS as differentially expressed at four different time points after *E. tenella* sporozoites infection of cell monolayers (4, 24, 36, and 48 hpi). From these identified proteins, we selected those described as being related to invasion and endogenous development and clustered them according to level of expression at the different time points (Figure 4). They grouped into three main distinct clusters: proteins expressed in freshly-released sporozoites (ready for invasion) which are downregulated after invasion and during development (Figure 4B); proteins overexpressed early after invasion (Figure 4A); proteins overexpressed during schizont development and/or in fully-formed fist-generation merozoites (Figure 4C).

There was very good correlation between the identified proteins expression along the time course and what has been described in the literature. Sporozoites ready to invade show overexpression of five different surface antigens (SAGs) which are important proteins in the recognition and attachment of the host cell (Tabares et al., 2004), as well overexpression of apical membrane antigen-1 (AMA1) and three rhoptry neck proteins (RONs) which are components of the moving junction, an essential structure for the invasion of the host cell in many Apicomplexa parasites (Besteiro et al., 2011). Three rhoptry kinases (ROPK), essential for intracellular survival through interaction with host proteins (Diallo et al., 2019), are also overexpressed by these pre-invading sporozoites. Once sporozoites have invaded the host cell (4 hpi), a switch in repertoire of SAG protein expression was observed, with four different SAGs found overexpressed here. All the detected microneme proteins (MICs), which are secreted and re-distributed onto the sporozoite surface to establish physical contact with the host cell during invasion (Carruthers and Tomley, 2008) are overexpressed in this cluster, indicative of recent and active invasion that is still taking place at this point (see Figure 1A). A rhomboid proteinase, involved in the cleavage of MICs from the parasite surface during invasion (Zheng et al., 2014), was also found. Sporozoite antigen SO7 and Eimepsin, proteins stored in refractile bodies—the largest sporozoite organelles that are of unknown function (de Venevelles et al., 2006), were also overexpressed at this point. Another different set of SAG proteins were overexpressed in the third cluster (corresponding to late development and/or fully-formed first-generation merozoites), together with two proteins homologous to dense granule proteins (GRAs) of other coccidians but not studied in *Eimeria* parasites; with functions are related to the formation of the parasitophorous vacuole membrane for intracellular survival (Mercier and Cesbron-Delauw, 2015).

### Parasite Replication and Stage-Specific Gene Transcription Evaluated by qPCR and RT-qPCR Evidenced the Endogenous Development Previously Observed by Microscopy

**Figure 5:**
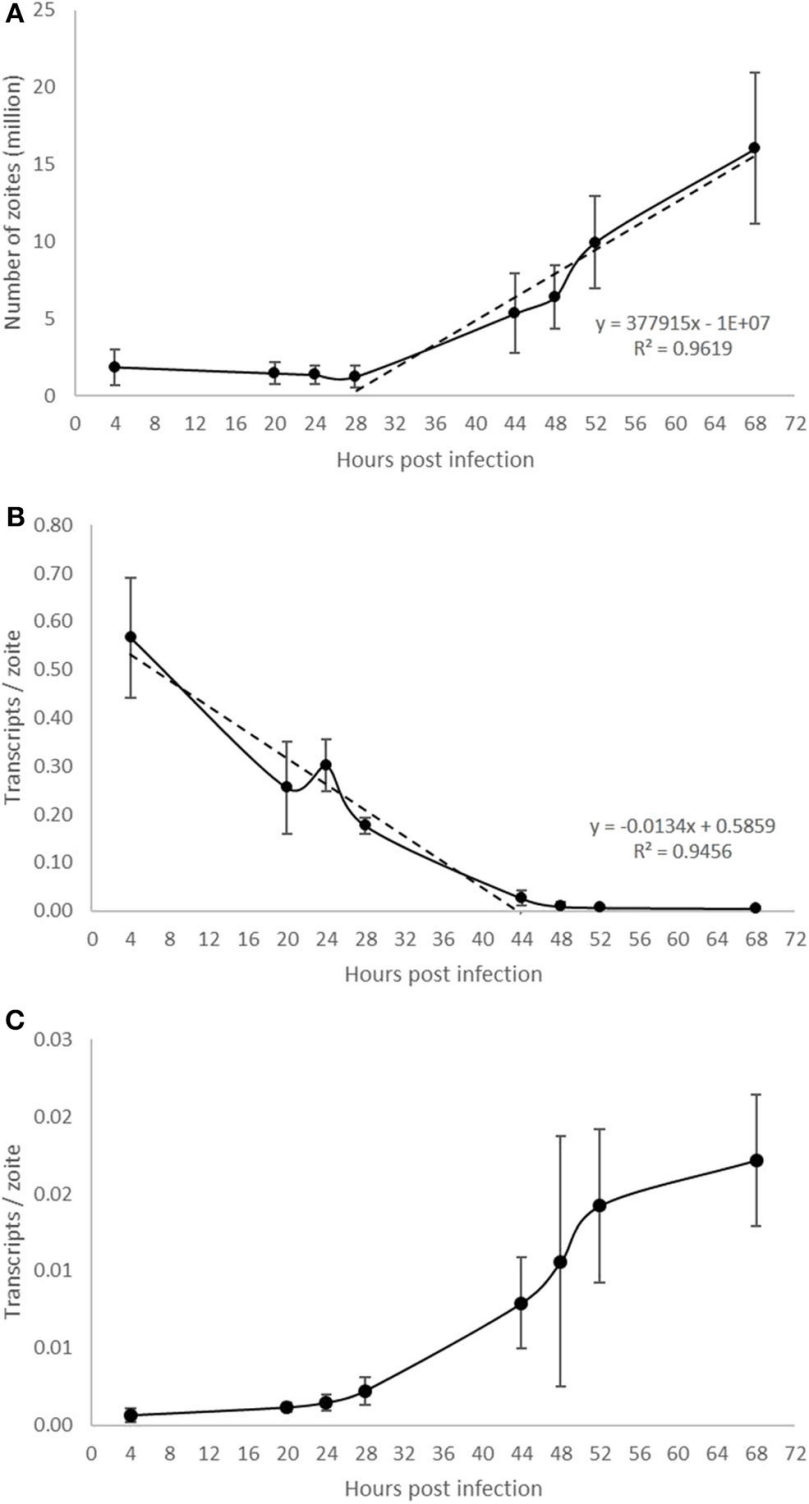
Evaluation of *E. tenella* endogenous development *in vitro* by qPCR and RT-qPCR. **(A)** DNA replication of the parasite commences around 28 hpi and increases in a linear fashion throughout the whole sampling time-course. **(B)** The number of transcripts per zoite corresponding to sporozoite-specific target SP25 decreases during first generation schizogony and is barely detectable after 44 hpi. **(C)** The number of transcripts per zoite corresponding to merozoite-specific target MZ25 increases during schizogony, starting before the onset of DNA replication at 28 hpi and continuing in a linear fashion until around 52 hpi when the first released merozoites were seen in cultures (see Figures 1P–R). Bars = SD.

Parasite development was analyzed in a time course experiment by qPCR (Figure 5). Increasing amounts of parasite DNA were detected from 28 hpi onwards (indicative of nuclear replication; Figure 5A) with levels increasing in a linear fashion thereafter (with a doubling time of 13.2 h). To determine if we could detect changes in parasite endogenous gene expression earlier in the process of schizogony (before the onset of nuclear replication detected by qPCR) we targeted two abundant stage-specific genes (by RT-qPCR). Transcripts of the sporozoite specific gene product SP25 showed a linear decrease in abundance immediately after infection (Figure 5B), before nuclear division started, and were almost undetectable from 44 hpi onwards. In contrast, transcripts of the merozoite specific gene product MZ80 increased slowly during the initial 28 hpi and thereafter increased rapidly until around 52 hpi (Figure 5C). These later time points show high variability due to the asynchronous nature of development in addition to the schizont rupture and merozoite release observed at these time points (see Figures 1P–R). The transcription of EtActin remained at a low and constant level throughout schizogony, as reported previously (Marugan-Hernandez et al., 2017a). We conclude that measuring the transcription of these two stage-specific gene products provides a useful assay for detecting changes to parasite metabolism that can be measured prior to DNA replication or schizont visualization. SP25 and MZ80 normalized RT-qPCR datasets showed a strong negative correlation (*r* = −0.929) which fits perfectly with the decreased numbers of sporozoites in the cultures at 28 hpi and the appearance of immature then maturing schizonts. Wild type parasites of *E. tenella* and transgenic *E. tenella* YFPmYFP displayed equivalent replication rates and levels of transcription (data not shown).

### ImageJ Semi-Automated Analysis Simulated the Endogenous Development

**Figure 6:**
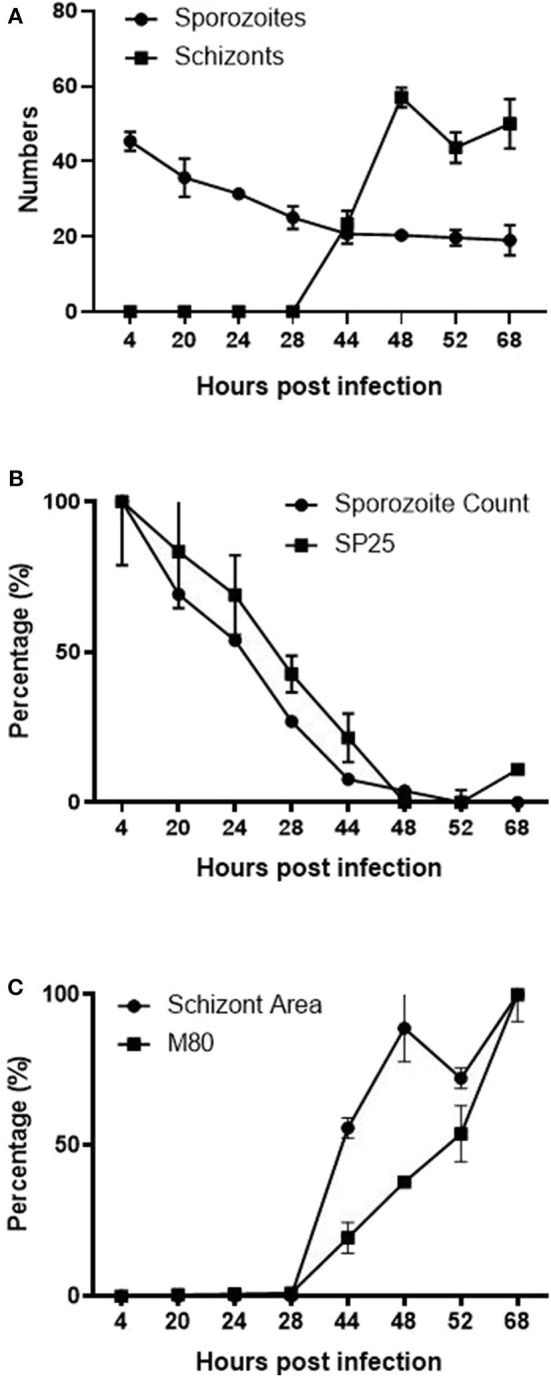
Correlation between ImageJ semi-automated analysis and qPCR data. **(A)** Graph showing the normalized number of fluorescent (YFP expression) sporozoites and schizonts detected by semi-automated image analysis over time. **(B)** Normalized data showing the number of sporozoites and SP25 gene transcription over time. **(C)** Normalized data comparing the average size of schizonts and MZ80 gene transcription over time.

Computational analysis of photographs taken via microscopy strongly supported the visual observations (Figure 1). Normalized datasets for sporozoite and schizont numbers taken from semi-automated image analysis were shown to have a strongly negative correlation (*r* = −0.855). In the first 28 hpi, the number of sporozoites gradually decreases as schizont development begins, reaching a plateau at 44 hpi (Figure 6A). Conversely, the numbers of schizonts sharply rises between 28 and 48 hpi contributing to this correlation pattern. A drop in schizont numbers is seen at 52 hpi, which correlates with a decrease in the overall measured schizont area at this time, which is attributed to the rupture of fully mature schizonts releasing merozoites (Figures 1P–U) and the decrease of YFP expression caused by the EtMIC1 promoter. A remaining base level of sporozoites can be seen until the end of the experiment which can be attributed to intracellular sporozoites that fail to develop.

Normalized transcript levels by RT-qPCR for SP25 gene and sporozoite numbers from semi-automated analysis were shown to have a very strong positive correlation (*r* = 0.979). SP25 transcription values fell between 4 and 44 hpi (Figure 5B) as sporozoites invaded the host cells and began schizogony (Figures 1J–L). These values were supported by the numbers of sporozoites documented during image analysis, which mirrored these changes (Figure 6B). Normalized MZ80 transcripts and schizont area data were shown to have a strong positive correlation (*r* = 0.913) (Figure 6C). The steady increase in MZ80 transcription beginning from 28 hpi onwards is shortly followed by the appearance of small schizonts at 44 hpi. Schizont growth and MZ80 gene transcription continued in a linear fashion until 52 hpi, when a drop in mean schizont area size can be seen with no comparative effect on MZ80 transcription. These findings can be attributed to the presence of newly-released first-generation merozoites emerging at 52 hpi from ruptured schizonts (Figures 1P–R), allowing MZ80 levels to be detected but not ruptured schizonts.

### The Effects of Anticoccidial Drugs on Invasion and Endogenous Development Can Be Assessed by qPCR *in vitro*

**Figure 7:**
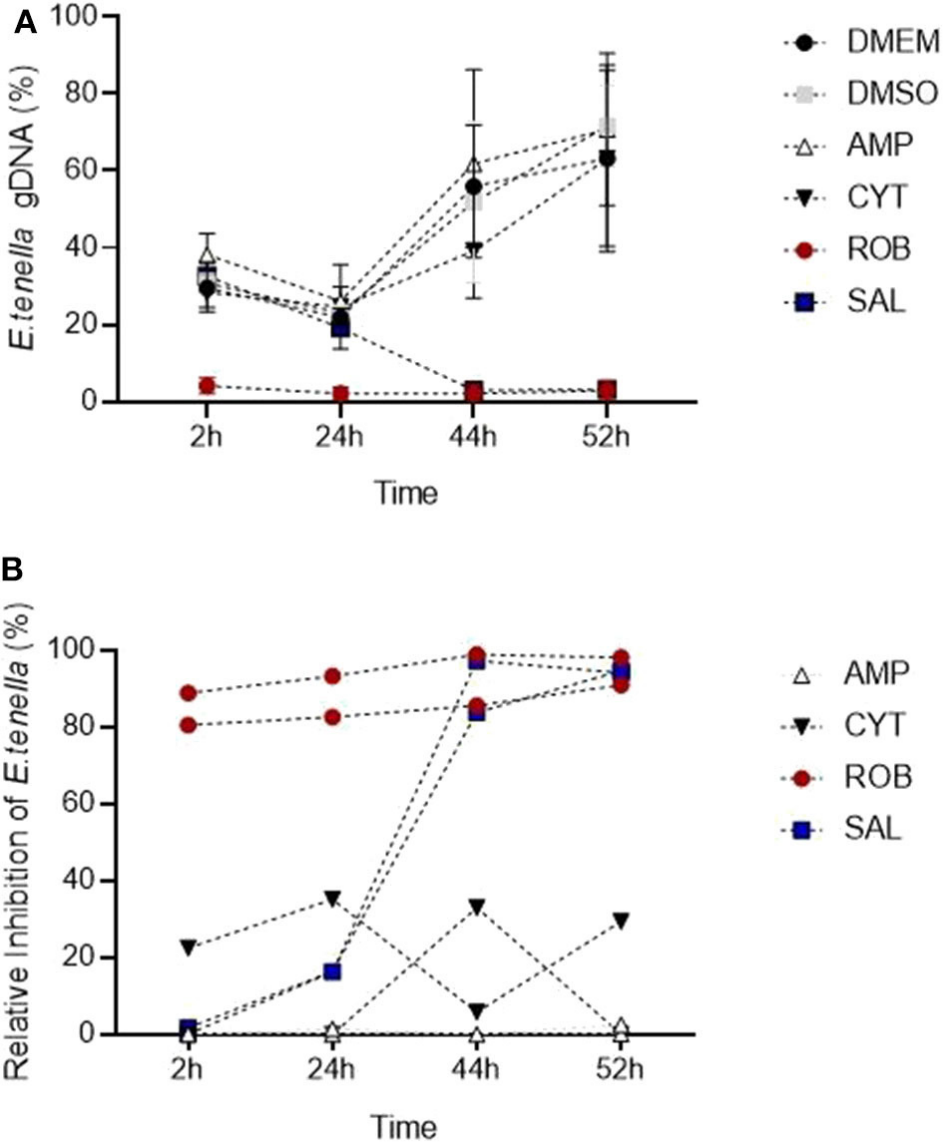
Detection of changes in invasion and endogenous development of *E. tenella* by qPCR when sporozoites are pre-incubated with anticoccidial drugs (5 μg/ml). **(A)** Time course showing the impact of drugs: robenidine (ROB) affected sporozoite invasion; salinomycin (SAL) allowed invasion but not development (parasite could have potentially died after invasion); amprolium (AMP) and cytochalasin D (CIT) did not have any effect at the tested concentrations (potentially caused by a reversible effect after the drug was removed). Bars = SD. **(B)** Relative inhibition of each drug (in percentage) in invasion and development for each time point when normalized to DMSO controls. Each line correspond to an individual experiment.

The *in vitro* system for *E. tenella* followed by qPCR analysis was suitable to detect the effect of different anticoccidial drugs on sporozoite invasion and development in a time course experiment (Figure 7A). Early after sporozoites were added to MDBK monolayers (2 hpi), sporozoites pre-incubated with robenidine showed a significant reduction of invasion compared to the control (Figure 7A; ROB vs. DMSO, Dunnett's multiple comparisons test) but no effect was seen with any of the other treatments. At later points of *Eimeria* endogenous development (44–52 hpi), a significant reduction in schizont area was seen in samples incubated with salinomycin (Figure 7A; SAL vs. DMSO, Dunnett's multiple comparisons test). No significant effects were seen in samples treated with amprolium (AMP) or cytochalasin D (CYT).

To assess the level of inhibition caused by pre-incubation with each drug, the proportion of parasites for each time point was also evaluated normalizing values of treated samples to their DMSO control (Figure 7B; Thabet et al., 2017). Proportions showed that amprolium caused the lowest effect against the invasion and development with a maximum inhibition of 1.3%. In contrast, a high effect (> 80%) was seen for robenidine from 2 hpi to the end of the time course. There was also an inhibition after exposition of parasites to salinomycin and cytochalasin D until 44 hpi, however the proportion of parasites increased again for this last group until 52 hpi.

In summary, pre-incubation with 5 μg/ml with amprolium or cytochalasin did not affect the invasion and development, probably due to a reversible effect after the removal of these drugs. Pre-incubation with salinomycicn did not affect invasion but inhibited further development, potentially caused by sporozoites dying early after invasion. Finally, pre-incubation with robenidine strongly affected invasion and therefore no further development was possible. The significant interaction between the specific anticoccidial drug and the time point supported that the development of *E. tenella* is dependent of both factors (drug and time) (Two-way RM ANOVA test, *p* < 0.001).

## Discussion

The use of *in vitro* models has been paramount for the study of protozoan parasites. The ability to replicate behaviors in a controlled environment has allowed the study of specific mechanisms and/or treatments in isolation of other factors, validating hypotheses and/or formulation of new ones to be verified later by *in vivo* models. The availability of *in vitro* models for apicomplexan parasites is limited and usually restricted to only one or a few stages of their complex life cycles (Muller and Hemphill, 2013). In heteroxenous Sarcocystidae coccidia (e.g., *Toxoplasma gondii, Neospora caninum*, or *Besnoitia besnoiti*) the tachyzoite stage responsible for the organic dissemination of the parasite in intermediate hosts can be continuously grown *in vitro* and this has advanced our understanding of many cellular mechanisms in apicomplexan parasites (Boothroyd, 2020). For monoxenous Eimeriidae coccidia (e.g., *Eimeria* species) there is no efficient *in vitro* system supporting continuous replication of a single stage or the completion of the lifecycle and much research in these species relies on the use of animals. This significantly limits studies especially for understanding the whole endogenous lifecycle including both asexual and sexual phases. A recent study revealed that successful endogenous development and production of small numbers of sexual stages can be achieved in the immortalized CLEC-213 chicken lung epithelial cell line; however, no oocysts were observed (Bussiere et al., 2018).

There are several immortalized cell lines that can support *Eimeria* sporozoite infection and first generation schizogony. In a comparative analysis Tierney and Mulcahy (2003) concluded that MDBK cells were most suitable, and this has been the line of choice for research in *E. tenella* for many years (Crane et al., 1984). Using this *in vitro* MDBK system we observed fully formed first-generation merozoites that exhibit the same structures as those described previously for first-generation merozoites generated in chick kidney cells (Pacheco et al., 1975). Nonetheless, as has been reported previously, these merozoites did not reinvade MDBK cells, for reasons that are not clear but which seem to represent a bottleneck to completing the full lifecycle *in vitro*, since second-generation merozoites obtained from chickens are capable to continue development when added to a monolayer of cultured cells (Xie et al., 1990). Another interesting observation is the apparent co-ordinated nuclear division seen in the three schizonts imaged by SBF-SEB; nonetheless further data would be necessary for the verification of this hypothesis.

Additionally, the MS identified a series of proteins directly related to invasion and development which are differentially expressed at specific time points in an *in vitro* time course. Results correlated with previous findings for some of these proteins: (i) presence of alternative types of surface antigen proteins (SAGs) between sporozoites and merozoites (Tabares et al., 2004); (ii) high abundance of microneme (MIC) proteins during invasion (Bumstead and Tomley, 2000); and (iii) overexpression of rhoptry (ROP) and dense granule (GRA) proteins during endogenous development (Oakes et al., 2013; Xue et al., 2020). These data further corroborate the suitability of the *in vitro* system to mimic endogenous development (Burrell et al., 2020) and are an important source of information to design specific interventions for the study of these relevant proteins. The detection by MS of these proteins indicated that they are present at significantly abundant levels to be targeted (e.g., antibody binding, enzymatic cleavage) and evaluated by this *in vitro* system.

*Eimeria* parasites undergo asexual replication by the process of schizogony (Pacheco et al., 1975). The beginning and end products of each round of multiplication are host cell invasion-competent “zoites” (sporozoite or merozoite). Between ~30 and ~48 h post-invasion intracellular parasites undergo multiple rounds of DNA replication and mitosis, followed by cytoplasmic expansion and budding of new zoites at the plasma membrane of the enlarged schizont from ~ 48 and ~72 h (Pacheco et al., 1975; Ferguson et al., 2008). This complex process can be monitored in cell culture by counting numbers of intracellular sporozoites, immature/mature schizonts, and released merozoites. Since early schizonts are not easily visualized by bright field, monolayers require fixation and staining (Brown et al., 2000) to perform counts. This is labor-intensive and may be subjective, especially at later times where there is asynchronous growth. Higher throughput quantification of parasite DNA replication can be achieved by measuring [3H] uracil incorporation into acid-insoluble material (Pfefferkorn and Pfefferkorn, 1977). Use of qPCR to count parasite genomes in newly invaded or developing cell cultures has been also reported recently (Jenkins et al., 2014; Thabet et al., 2015). Since the use of qPCR is as reliable as traditional methodologies to quantify parasites in chickens (Nolan et al., 2015) we evaluated its suitability to reflect the changes observed by microscopy in this *in vitro* system. To avoid time-consuming staining of samples, we used a fluorescent population of *E. tenella* previously generated by transgenesis (Clark et al., 2008), which allowed the direct visualization of schizonts and a reduced number of samples (micrographs can be taken at different time points from the same sample). Furthermore, to avoid subjectivity, we used semi-automated image analysis by the generation of an ImageJ plugin to provide quantitative data from the images. A perfect correlation was found between visual observations, qPCR data and image analysis whereby there were no significant changes before the 28 hpi sampling point and thereafter both DNA content and total schizont area increased in a linear fashion until 54 hpi. Therefore, both ways of quantification are valid to predict endogenous development of *E. tenella* in MDBK cells.

Neither of the two methods for quantification detected changes before the 28 hpi time-point. We hypothesized that quantifying stage-specific gene transcription from cDNA could provide strong markers and a more robust method to monitor parasite progression through the early and late stages of schizogony through to release of merozoites, which cannot be done using qPCR alone. Although there are no published reports on the timing of merozoite-specific gene transcription during first generation schizogony in cell culture, from analysis of the *E. tenella* genome by Reid et al. (2014) and consideration of the accompanying RNAseq database (ToxoDB) we selected genes that were suitable for monitoring endogenous development based on previous experiments performed in our group (unpublished). SP25 is expressed only in sporozoites whereas MZ80 is merozoite specific. Both are members of a novel gene family that is found only in the *Eimeria* genus and for which there is currently no ascribed biological function (*esf2* family; Reid et al., 2014). During first generation schizogony the number of SP25 transcripts decreased rapidly between 4 and 44 hpi reaching a level that was close to undetectable. In contrast, transcripts for MZ80 were detected at a low level until 20–24 hpi and thereafter increased rapidly until 52 hpi, which coincides with the start of merozoite release. We consider therefore that the reduction of SP25 transcription could be a marker to predict changes at early time points (without the need of performing a longer time course) whereas MZ80 a marker for late schizogony and merozoite formation.

Once the *in vitro* system was well-characterized by quantitative methods, we used compounds with known effects as anticoccidials to test how sensitive these methods were in detecting changes in sporozoite invasion and schizont development. We used a protocol in which sporozoites were pre-treated for 1 h and then drugs were removed before adding them to the cell monolayer. This was to simplify the assay and avoiding having to perform cytotoxicity tests for each drug. In addition, unpublished results showed that DMSO, the usual solvent for drugs, affected *Eimeria* development when added to the monolayer at 0.05% compared to the untreated control (data not shown). Therefore, pre-treatment was considered a better way to test the *in vitro* model to detect changes in invasion/development if drugs are prepared in DMSO; an alternative solvent would be recommended for a direct treatment onto the cell monolayer.

Amprolium, a thiamine antagonist with recorded peak activity against maturing first generation schizonts (McDougald and Galloway, 1976), did not show any effect on sporozoite invasion and schizont development, which is likely to be because of the early removal of the treatment, several hours before this peak of action. We did not detect a reduction of invasion when pre-treating with cytochalasin D, an inhibitor of actin polymerization that blocks movement and in consequence reduced sporozoite invasion (Bumstead and Tomley, 2000). Since the action of this drug is reversible, we assume that removal of the treatment allowed parasites to infect cells normally. By contrast robenidine, a guanidine derivative which inhibits oxidative phosphorylation, had a strong effect on sporozoite invasion which was clearly reflected by the qPCR data. McDougald and Galloway (1976) did not find these same effects, but in their study treatment was added at the time of invasion and at a lower dose (0.01 μg/ml) to what was used here. Microscopic observations of lack of movements and changes in the morphology of sporozoites pre-exposed to robenidine suggests that the low amount of gDNA we found throughout the time course was due to early death of the parasites. Finally, the progressive reduction from 24 hpi in parasite gDNA and transcripts following salinomycin treatment indicates that sporozoites were able to invade cells, being affected only during further development. We hypothesize that treatment with this ionophore damaged sporozoite membranes allowing parasite invasion to occur but inducing death thereafter since there is no previous evidence that ionophores can affect intracellular stages (Mehlhorn et al., 1983; Thabet et al., 2015). The latter authors used a similar method to calculate inhibition/development based on qPCR and showed that treating sporozoites for 2 h with 1 μg/ml of salinomycin did not affect invasion of MDBK cells whereas significant inhibition was found if sporozoites were treated for 4 h. In this same study, inhibition assays using the same concentration of salinomycin reduced sporozoite replication to 50% after 24 hpi and by 95% after 48, 72, or 96 hpi. Our results agree with those of Thabet et al. (2015) showing a time-dependant and a progressive effect of salinimycin in the later stages of development.

In conclusion, in this study we have validated the suitability of the MDBK cell culture *in vitro* system to mimic the *in vivo* behavior of sporozoite invasion and first generation schizogony of the endogenous *E. tenella* lifecycle. We have described quantitative methods for use in assessing the effects of anticoccidial drugs and novel compounds against parasite invasion and asexual development that can support a guiding principle for the ethical use of animals in sciences, reducing their use in *Eimeria* research.

## Data Availability Statement

The raw data supporting the conclusions of this article will be made available by the authors, without undue reservation.

## Ethics Statement

The animal study was reviewed and approved by Royal Veterinary College Ethical Review Committees and the United Kingdom Government Home Office.

## Author Contributions

VM-H and FT conceived and designed the project. AB and SV performed the TEM and SBF-SEM. VM-H, DX, and NR generated the samples and MS data. VM-H standardized the qPCR and RT-Qpcr and wrote the manuscript. GJ run qPCR and RT-qPCR and developed the ImageJ plugin for semi-automated image analysis. KA-M performed the anticoccidial drug assays and analyzed the data. FT and SV critically reviewed and edited the paper. All authors read and approved the final version of the manuscript.

## Conflict of Interest

The authors declare that the research was conducted in the absence of any commercial or financial relationships that could be construed as a potential conflict of interest.
